# Morbidity in Elderly Women Undergoing Pelvic Floor Reconstruction

**DOI:** 10.7759/cureus.100361

**Published:** 2025-12-29

**Authors:** Kay T Win, Yahia Refaee, Jaydip Dasgupta, Bivas Biswas, Bhawana Purwar

**Affiliations:** 1 Department of Obstetrics and Gynaecology, University Hospitals of Derby and Burton NHS Foundation Trust, Derby, GBR

**Keywords:** elderly women, morbidity, pelvic floor reconstruction, pelvic organ prolapse, quality improvement, urogynaecology

## Abstract

Introduction: Pelvic floor reconstruction (PFR) in elderly women remains underutilised due to perceived surgical risk. With increasing life expectancy and functional demands, it is essential to evaluate contemporary outcomes in this population. This study aimed to review the perioperative morbidity and short-term outcomes of PFR in women aged ≥70 years and to contextualise results against recognised clinical benchmarks. Methods: We conducted a single-centre retrospective cohort review of all women aged ≥70 years who underwent PFR at Royal Derby Hospital (RDH) between September 2022 and August 2024 (n=86). Patient demographics, comorbidities, risk factors, type of procedure, complications, and recurrence within one year were analysed. Functional outcomes were assessed using patient-reported symptom status informed by International Consultation on Incontinence Questionnaire (ICIQ) documentation, with analysis focused on completion rates and categorical postoperative symptom outcomes. Complication rates were presented with 95% confidence intervals (CIs) and contextualised against national benchmark data to evaluate the quality of care and surgical safety. Statistical analyses were descriptive and exploratory.

Results: The mean age was 77.3±5.2 years, and the mean body mass index (BMI) was 27.8±4.3 kg/m², with 36% (n=31) classified as overweight. Most patients were multiparous (median parity 3, range 1-7). Hypertension (68.6%, n=59), constipation (45.3%, n=39), musculoskeletal disorders (38.4%, n=33), diabetes (32.6%, n=28), respiratory disease (27.9%, n=24), cardiac disease (25.6%, n=22), endocrine disorders (16.3%, n=14 ), and mental problems (10.5%, n=9 ) were the most prevalent comorbidities. Combined procedures, such as vaginal hysterectomy with anterior/posterior repair ± sacrospinous fixation, were performed in 84.9% of cases (n=73), while 15.1% of cases (n=13) involved isolated procedures, including four cases of colpocleisis in patients with multiple comorbidities and no requirement for sexual function. No intraoperative complications occurred. Postoperative morbidity was low: Urinary or wound infection occurred in 4.6% (95% CI 1.8-11.4%), readmission within 30 days in 4.6% (95% CI 1.8-11.4%), and ileus, failed trial without catheter (TWOC), vaginal adhesion, and persistent postoperative pain each in 1.1% (95% CI 0.2-6.3%). No cases required return to theatre or resulted in death (95% CI 0.0-4.3%). Symptomatic improvement was reported by 72.1% (95% CI 61.8-80.5%, n=62) of patients, while 17.4% (95% CI 10.9-26.8%, n=15) had persistent and 10.5% (95% CI 5.6-18.7%, n=9) developed new urinary urgency or frequency. One unrelated death occurred within five months after surgery.

Conclusion: PFR in women aged ≥70 years appears to be safe and associated with favourable short-term morbidity and patient-reported outcomes in appropriately selected, well-optimised patients when performed in a structured multidisciplinary urogynaecology service. Observed complication rates were low and comparable to national benchmarks, supporting surgical management following individualised assessment and optimisation. However, given the exploratory design and limited follow-up, results should be interpreted cautiously, and prospective multicentre studies incorporating frailty assessment and longer-term follow-up are required to evaluate durability, recurrence, and quality-of-life outcomes.

## Introduction

Pelvic organ prolapse (POP) is a common disorder affecting one in 10 women over the age of 50 years, and significantly affects women's quality of life [1]. It occurs when the pelvic floor muscles and connective tissues that support the bladder, uterus, and rectum become weakened or stretched, resulting in the descent of these organs into or beyond the vaginal canal [1-3]. This disruption of normal pelvic anatomy may lead to a variety of symptoms, including a sensation of pelvic pressure, vaginal bulge, urinary or bowel dysfunction, and sexual discomfort. These symptoms can cause both physical inconvenience and emotional distress, limiting mobility, social participation, and sexual well-being.

The likelihood of developing POP increases with advancing age, parity, menopause, and conditions that chronically increase intra-abdominal pressure, such as obesity, constipation, or chronic cough [1-3]. Declining collagen and oestrogen deficiency reduce tissue elasticity, increasing susceptibility to prolapse and impaired healing. The global prevalence ranges from 3% to 50% depending on definitions and the population studied [4-7]. As life expectancy increases and more women live longer with chronic comorbidities, the number of older women living with symptomatic POP and requiring intervention is expected to rise, placing additional demand on urogynaecology services [4-7].

As the UK population ages, the proportion of women over 70 years seeking surgical management for prolapse continues to rise. Traditionally, elderly patients were considered high-risk surgical candidates due to comorbidities and concerns about postoperative recovery. However, contemporary data indicate that, with appropriate patient selection and perioperative optimisation, surgical outcomes in elderly women are comparable to those in younger cohorts [8-10]. Several series of women aged 70-80 years and above undergoing vaginal or laparoscopic pelvic floor reconstruction (PFR) have reported low perioperative mortality and high satisfaction, although sample sizes are often modest and inclusion criteria are selective [8-12].

Emerging evidence also suggests that frailty and functional status, rather than chronological age alone, are key determinants of perioperative risk and recovery in this population. Studies using comorbidity indices and frailty measures demonstrate that higher frailty scores are associated with increased complications and prolonged hospital stay after prolapse surgery. At the same time, well-optimised elderly women can achieve outcomes comparable to younger, less comorbid patients [10,13]. These findings support an individualised approach to surgical decision-making that incorporates geriatric assessment and functional reserve alongside chronological age.

Despite this evolving evidence base, important gaps remain. Most published cohorts either combined older and younger women or focused on very elderly subgroups without detailed benchmarking against national quality standards. Long-term durability of POP surgery has been described predominantly in mixed-age populations [12], and contemporary UK data describing early safety, perioperative morbidity, and patient-reported symptom improvement specifically in women aged ≥70 years are limited. In particular, there is a paucity of reports that integrate the British Society of Urogynaecology (BSUG) benchmark indicators with validated patient-reported outcome measures (PROMs) such as the International Consultation on Incontinence Questionnaire (ICIQ), within a structured quality-improvement framework.

At Royal Derby Hospital (RDH), the urogynaecology department has observed a gradual increase in the number of women aged 70 years and above referred for PFR procedures for POP. This demographic trend underscores the importance of continually evaluating surgical outcomes in this population to ensure care remains effective, safe, and patient-centered. Monitoring postoperative morbidity, recurrence rates, and quality-of-life improvement allows services to benchmark their outcomes against national standards and maintain high-quality care.

This quality improvement project was therefore undertaken to address these gaps by evaluating the contemporary outcomes of PFR in women aged 70 years and above within a UK tertiary urogynaecology service. The primary objective was perioperative morbidity, defined by postoperative complications and 30-day readmission rates, and benchmarked against data from the BSUG national database [14]. The secondary objective included early PROMs, assessed using the ICIQ, as well as persistent or recurrent symptoms during short-term follow-up. Outcomes were assessed during the early postoperative period up to 30 days for complications and at the first postoperative clinic review for functional outcomes in four months' time, and then recurrence within a period of one year after surgery. By focusing on a clearly defined elderly cohort within a contemporary UK tertiary urogynaecology service, and by integrating both clinical outcomes and PROMs, this study seeks to provide pragmatic, real-world evidence on the safety and early effectiveness of PFR in appropriately selected older women, and to inform local service development and wider clinical practice. This study was designed as an exploratory retrospective cohort review, aiming to describe safety and early effectiveness in a carefully selected elderly population rather than to test a predefined causal hypothesis.

## Materials and methods

Study design and setting

A retrospective cohort review was conducted of women aged ≥70 who underwent PFR at RDH between September 2022 and August 2024. The project was registered as a Quality Improvement Project with the Clinical Governance Department (Audit ID available upon request).

Inclusion and exclusion

Women aged ≥70 undergoing reconstructive surgery for POP were eligible for inclusion. Eligible patients were identified through a structured search of the hospital electronic theatre management system and urogynaecology operative logs. Procedures were screened using standard operative descriptors and coding for PFR procedures (including vaginal hysterectomy for prolapse, anterior and/or posterior vaginal wall repair, sacrospinous fixation, and colpocleisis). Identified cases were cross-checked against outpatient urogynaecology clinic lists and electronic patient records to confirm eligibility and completeness of data.

Exclusion criteria were operations for malignancy, mesh-related complications, or incomplete records. This ensured that outcomes reflected prolapse-specific surgical morbidity and symptom improvement within a clearly defined cohort.

Data collection

Electronic medical records, theatre logs, clinic letters, and discharge summaries were reviewed by two independent clinician authors, who entered information into a standardised data collection spreadsheet to minimise transcription error. Any discrepancies between reviewers were resolved by consensus. Missing data were assessed on a variable-by-variable basis and cross-checked against alternative sources, including clinic correspondence and operative notes, where available. Cases with incomplete key outcome data were excluded from relevant analyses; no imputation was performed, and all analyses were conducted using available-case methodology. A 10% random sample was independently validated by a senior consultant for accuracy.

Variables and outcomes

Baseline variables collected included demographics (age, parity, BMI, smoking), comorbidities (hypertension, diabetes, respiratory, cardiac, endocrine, renal, musculoskeletal, mental health, and constipation), preoperative use of vaginal oestrogen, and type of surgical procedure performed.

Postoperative complications were defined as adverse events occurring intraoperatively or within 30 days of surgery. Minor complications included urinary tract infection, wound infection, failed trial without catheter (TWOC), postoperative ileus, vaginal adhesion, or persistent pain managed conservatively. Infective complications were diagnosed based on documented clinical assessment and/or microbiological confirmation requiring antibiotic treatment. Failed TWOC was defined as the inability to void adequately following catheter removal, necessitating re-catheterisation. Readmission was defined as an unplanned hospital admission within 30 days of surgery related to postoperative morbidity. Major complications were defined as events requiring return to theatre, organ injury, intensive care admission, or resulting in death. Complication severity was interpreted in line with Clavien-Dindo classification principles, although formal grading was not prospectively assigned.

Postoperative follow-up data were obtained from routine outpatient clinic reviews, electronic patient records, discharge summaries, and clinical correspondence. Early postoperative outcomes were assessed at the first scheduled follow-up visit, typically occurring within four months after surgery. Postoperative complications and unplanned readmissions were captured within 30 days of surgery, in line with standard surgical audit practice. Assessment of symptom persistence or recurrence was undertaken during routine clinical follow-up and recorded for up to 12 months postoperatively. Outcomes beyond one year were not systematically available and were therefore outside the scope of this review.

Functional outcomes were assessed using patient-reported symptom status documented at routine postoperative review. Assessments were conducted and documented by a trained urogynaecology specialist nurse within the specialist urogynaecology team as part of standard clinical follow-up. Outcome assessment was not blinded but followed routine departmental practice. Significant symptomatic improvement was defined as patient-reported resolution or marked improvement of prolapse and/or urinary symptoms compared with the preoperative state, with patient satisfaction and no requirement for additional treatment. Persistent symptoms were defined as ongoing bothersome symptoms requiring continued conservative management. New symptoms referred to de novo urinary urgency or frequency reported postoperatively that were not present before surgery.

ICIQ forms were collected preoperatively and postoperatively; however, numerical ICIQ scores were not consistently transcribed into clinic documentation. Where available, ICIQ responses informed symptom assessment, but no predefined numerical ICIQ score thresholds or minimal clinically important difference (MCID) cut-offs were applied due to incomplete paired score capture.

Perioperative pathway

All patients underwent multidisciplinary preoperative assessment involving anaesthetists, medical teams, and specialist nursing staff. Optimisation strategies included management of hypertension and diabetes, bowel regulation, and preoperative vaginal oestrogen therapy for at least six weeks when clinically indicated. Formal frailty scoring systems were not routinely recorded during the study period. However, all patients underwent structured preoperative assessment by anaesthetists, including functional assessment and assignment of American Society of Anesthesiologists (ASA) physical status classification, which informed operative suitability and perioperative planning. Anaesthesia was delivered under general or regional techniques, as determined by anaesthetic assessment. Enhanced Recovery After Surgery (ERAS) principles were applied, including early mobilisation and standardised catheter management.

All procedures were performed by, or under the direct supervision of, consultant gynaecologists specialising in urogynaecology, with junior doctors or residents assisting in accordance with training requirements. Standardised departmental protocols were followed for operative technique and perioperative care.

Statistical analysis

As a retrospective quality-improvement study, no formal a priori sample size calculation was performed. The sample size reflects all eligible patients undergoing PFR within the predefined study period and was considered appropriate for an exploratory analysis of perioperative safety and early outcomes.

Statistical analyses were performed using IBM SPSS Statistics version 29 (IBM Corp., Armonk, NY). Descriptive statistics were used to summarise baseline characteristics and outcomes. Continuous variables are presented as means with ranges, and categorical variables as frequencies and percentages. Fisher’s exact test or two-proportion z-tests, chi-square tests, and McNemar’s test were applied where appropriate. 95% CIs were calculated for key complication rates. All statistical tests were two-tailed, with statistical significance defined a priori as p <0.05.

Outcomes were benchmarked against the BSUG national database, as it represents the largest UK-specific repository of PFR outcomes and is widely used for national audit, quality assurance, and service evaluation. Benchmarking was undertaken to contextualise local outcomes rather than for inferential comparison.

Patient-reported outcome analysis focused on completion rates and categorical postoperative symptom status, consistent with routine clinical documentation during the study period. Formal paired numerical ICIQ score change analysis and MCID calculations were not undertaken due to the incomplete availability of paired score data.

Data confidentiality and compliance

All data were anonymised before analysis, and no identifiable patient information was used or shared outside the Trust. Data handling complied fully with UK General Data Protection Regulation (GDPR) and NHS Digital Information Governance standards. As a registered Quality Improvement Project using routinely collected anonymised data, formal research ethics committee approval and individual consent were not required under NHS policy.

## Results

Demographic characteristics

**Figure 1:**
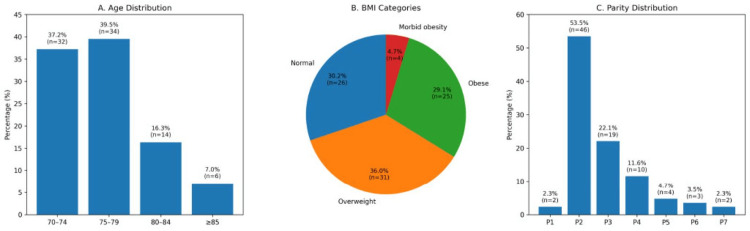
Demographic Characteristics of Women Undergoing PFR (n=86) (A) Age distribution of women undergoing PFR, demonstrating that the majority were between 70 and 79 years old. (B) BMI distribution based on WHO-derived categories: Normal weight (BMI <25 kg/m²), overweight (25-29.9 kg/m²), obese (30-39.9 kg/m²), and morbid obesity (≥40 kg/m²). Percentages and patient numbers (n) are shown for each category. (C) Parity distribution displayed as a bar chart, illustrating the proportion and number of women in each parity group. Values are expressed as percentages of the total study cohort (N=86). PFR, pelvic floor reconstruction.

In a total of 86 patients, the mean age of the participants was 77.3±5.2 years (range, 70-89 years), and the mean BMI was 27.8±4.3 kg/m² (range, 19.7-41.5 kg/m²). Most participants (76.7%) were aged between 70 and 79 years, and 36% were classified as overweight and 29.1% as obese, consistent with known risk factors for POP. These demographics are consistent with national BSUG registry data for women undergoing PFR in this age group [14]. The median parity was three (range 1-7), and most participants were multiparous, reflecting the demographic profile for pelvic floor dysfunction. Over half (53.5%) had parity of two, while nearly one-third had parity of three or more, consistent with established risk factors for the development of prolapse. The demographic characteristics of the study cohort are shown in Figure 1.

Comorbidities and risk factors

**Figure 2:**
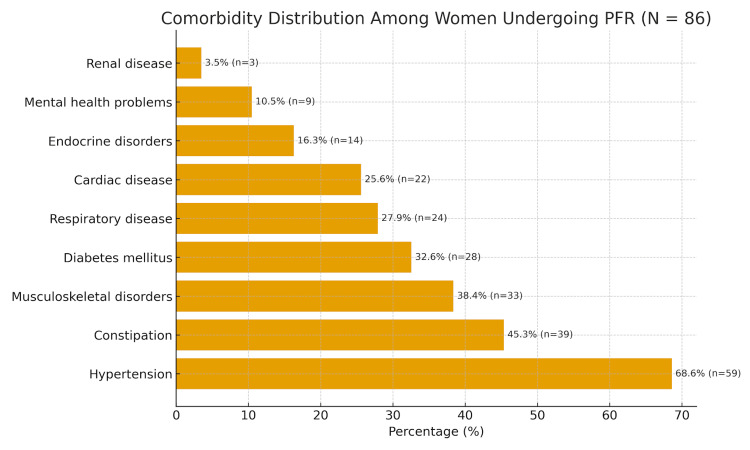
Comorbidities Among Women Aged 70 Years and Above Undergoing PFR (n=86) Individual comorbidities are shown with corresponding percentages and patient numbers. Hypertension, constipation, and musculoskeletal disorders were the most common, consistent with the expected health profile of elderly women undergoing PFR. Percentages are calculated from the total study cohort (n=86). PFR, pelvic floor reconstruction.

The distribution of comorbidities among the study is illustrated in Figure 2. Hypertension was the most prevalent comorbidity (68.6%, n=59), followed by constipation (45.3%, n=39), musculoskeletal disorders (38.4%, n=33), diabetes mellitus (32.6%, n=28), respiratory disease (27.9%, n=24), and cardiac disease (25.6%, n=22). Endocrine disease (16.3%, n=14), mental problems (10.5%, n=9), and kidney disease (3.5%, n=3) were less frequent. The high prevalence of hypertension and constipation underscores the need for perioperative optimisation and multidisciplinary care in elderly women undergoing PFR. Polypharmacy and mild frailty were common, but all patients were deemed fit for anaesthesia following multidisciplinary preoperative assessment.

Smoking was reported by only 12% of the participants, while 78% were on topical vaginal estrogen therapy preoperatively, reflecting proactive tissue optimisation [15].

Surgical profile

**Figure 3:**
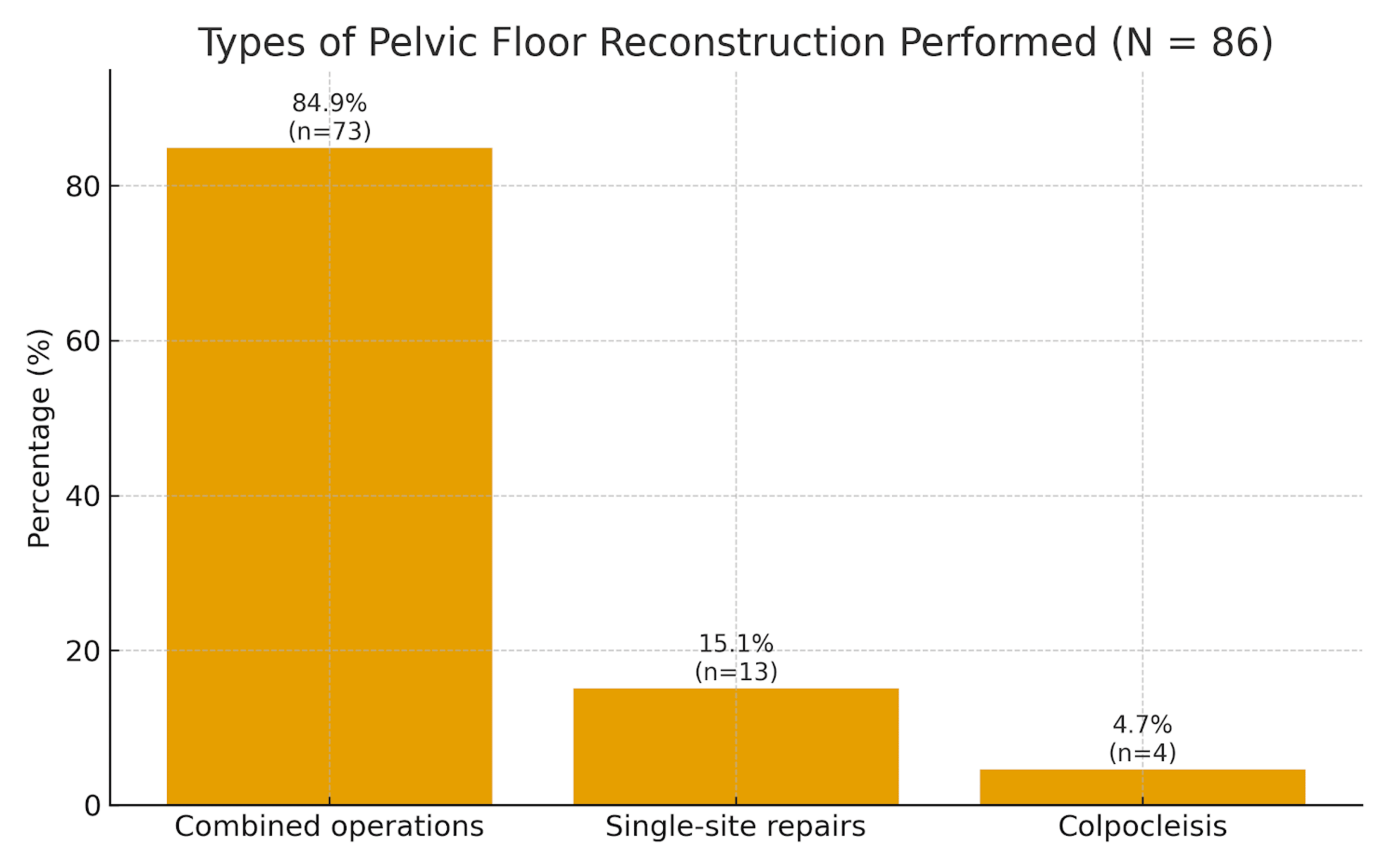
Types of PFR Performed Among Women Aged 70 Years and Above (n=86) Bar chart illustrating the distribution of surgical procedures. Combined operations accounted for the majority of cases, followed by single-site repairs and a small proportion of colpocleisis procedures, reflecting individualised surgical planning based on patient comorbidity and functional status. Percentages are calculated from the total study population (n=86). PFR, pelvic floor reconstruction.

The majority (84.9%, n=73) underwent combined operations: vaginal hysterectomy with anterior and/or posterior repair, with or without sacrospinous fixation. Thirteen women had single-site repairs (anterior or posterior repair or simple hysterectomy alone), and four women underwent colpocleisis due to non-sexual activity and multiple comorbidities. This pattern reflects standard practice in elderly populations, striking a balance between anatomical correction, surgical safety, and functional outcomes. The types of PFR procedures performed are presented in Figure 3.

Intraoperative complications

No intraoperative injuries to the bladder, ureter, or bowel occurred.

Postoperative complications

Postoperative morbidity was low: Urinary or wound infections occurred in 4.6% of patients (95% CI 1.8-11.4%), and 30-day readmission occurred in 4.6% (95% CI 1.8-11.4%). Failed TWOC, ileus, vaginal adhesion, and persistent postoperative pain each occurred in 1.1% of cases (95% CI 0.2-6.3%). No returns to theatre or surgery-related deaths were recorded (0%; 95% CI 0.0-4.3%).

When contextualised against BSUG benchmark data, observed rates were broadly similar; however, interpretation should be cautious, as this exploratory study is not powered to detect differences in rare outcomes. Given the relatively small sample size, these findings should be interpreted cautiously, as the study may be underpowered to detect modest but clinically meaningful differences in rare adverse events. Overall, the reporting of complication rates with confidence intervals and sensitivity analysis supports the robustness of findings within the constraints of an exploratory, retrospective cohort.

**Figure 4:**
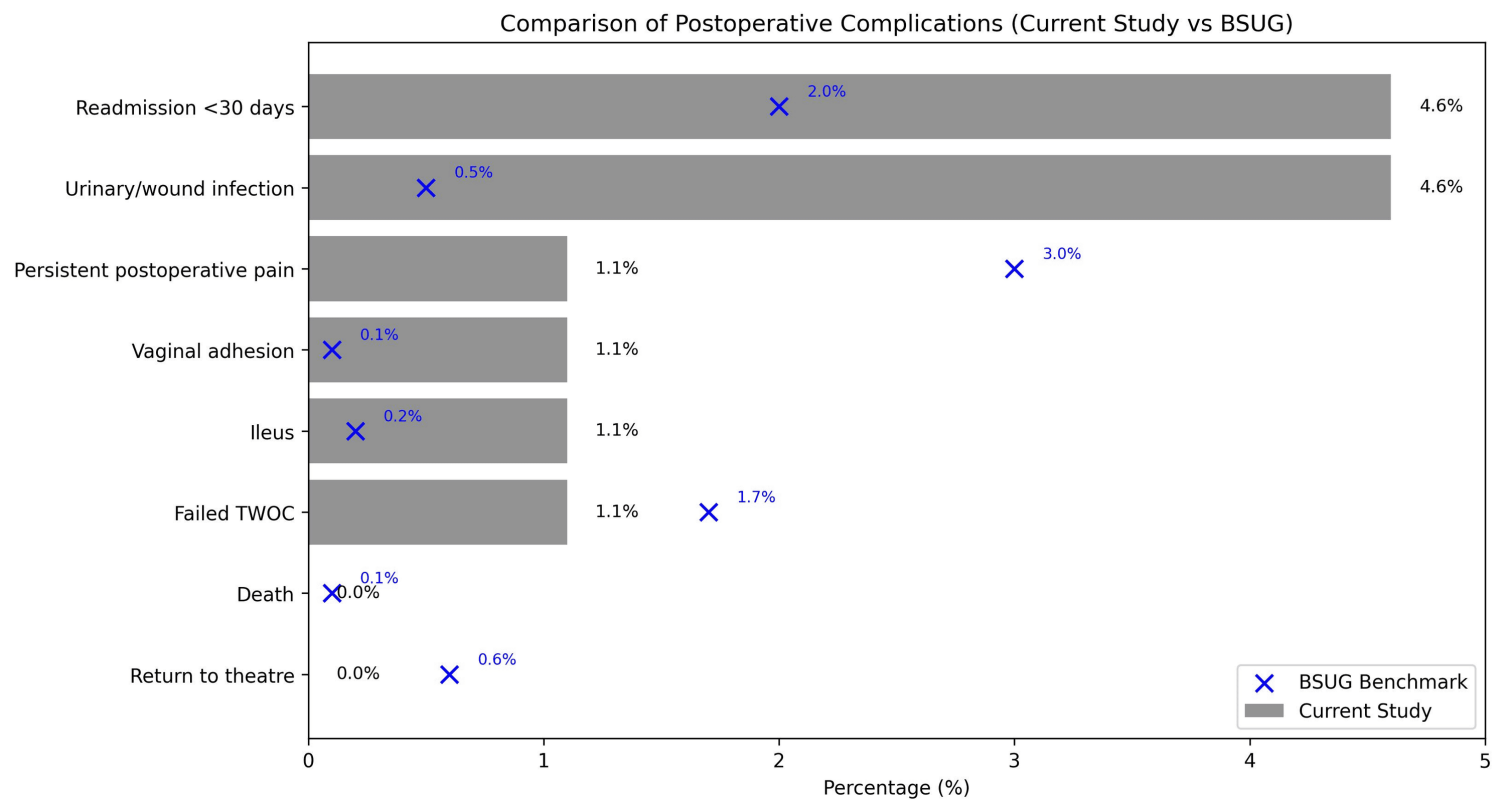
Comparison of Postoperative Complications Between BSUG Audit Database (2022) and RDH Data Horizontal bar chart showing postoperative complication rates observed in the present study (grey bars) compared with BSUG benchmark data (blue "x" marks). Data from BSUG National Database Annual Report (2022) were used for reference. TWOC, trial without catheter; BSUG, British Society of Urogynaecology; RDH, Royal Derby Hospital.

Postoperative complication rates are summarised in Table 1, and a visual comparison with BSUG averages is illustrated in Figure 4.

**Table 1:** Postoperative Complications Following PFR Compared With BSUG Benchmark Data (n=86) Observed complication rates are presented with 95% confidence intervals and contextualised against BSUG national audit benchmarks. No formal hypothesis testing was performed, as BSUG data are used for quality benchmarking rather than inferential comparison. TWOC, trial without catheter; BSUG, British Society of Urogynaecology; PFR, pelvic floor reconstruction.

Complication	n (%)	95% CI (lower-upper)	BSUG Benchmark (%)
Urinary/wound infection	4 (4.6%)	1.8-11.4%	0.50%
Readmission <30 days	4 (4.6%)	1.8-11.4%	2.00%
Failed TWOC	1 (1.1%)	0.2-6.3%	1.70%
Ileus	1 (1.1%)	0.2-6.3%	0.20%
Vaginal adhesion	1 (1.1%)	0.2-6.3%	0.10%
Persistent postoperative pain	1 (1.1%)	0.2-6.3%	3.00%
Return to theatre	0 (0%)	0.0-4.3%	0.60%
Death	0 (0%)	0.0-4.3%	0.08%

Functional outcomes

**Figure 5:**
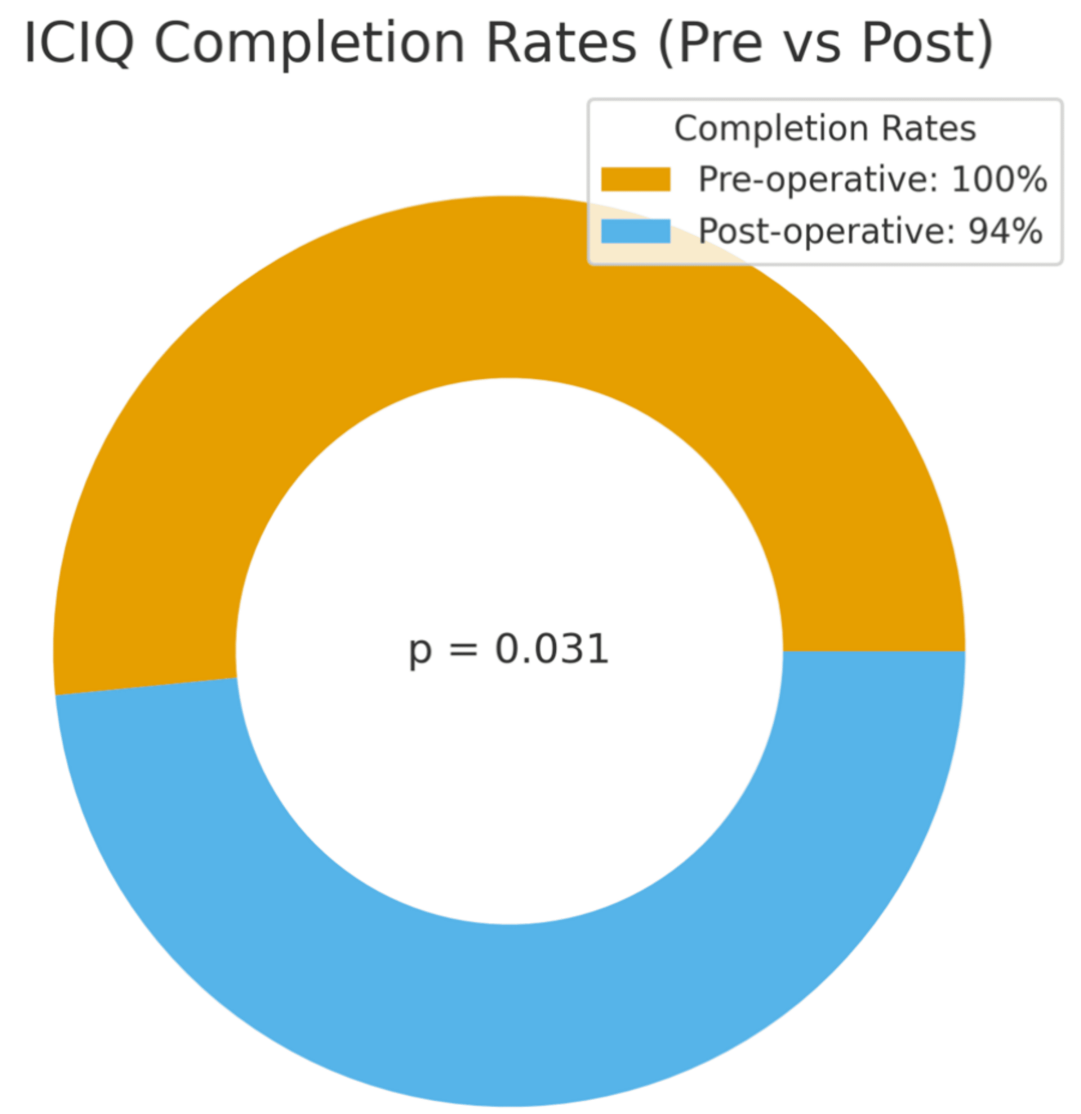
ICIQ Questionnaire Completion Rates Before and After PFR in Women Aged 70 Years and Above This donut chart demonstrates PROM compliance using ICIQ. Preoperative completion was 100% (86/86), while postoperative completion was 94% (80/86). McNemar's test revealed a statistically significant reduction in follow-up completion (p=0.031), although overall retention remained high, indicating strong patient engagement with postoperative quality-of-life assessment. PFR, pelvic floor reconstruction; PROM, patient-reported outcome measure; ICIQ, International Consultation on Incontinence Questionnaire.

Preoperative ICIQ compliance was 100% (n=86) compared to 94% (n=80) postoperatively (p = 0.031), indicating minimal loss to PROM follow-up due to other health conditions and demonstrating very high patient engagement (Figure 5). 

Because paired numerical ICIQ scores were incompletely captured, formal assessment of pre- to postoperative score change or MCID was not feasible. Accordingly, PROM results are presented descriptively. Sensitivity analysis was performed, excluding the six patients with missing postoperative ICIQ data. Among patients with complete paired questionnaires (n=80), the proportion of patients reporting significant symptomatic improvement remained largely unchanged, and the overall pattern of functional outcomes remained consistent. This suggests that missing PROM data did not materially influence the observed direction of functional improvement. Non-significant findings should be interpreted cautiously, as the study was exploratory and not powered to detect small differences or rare outcomes.

Formal POP-Q measurements and standardised clinical prolapse staging at follow-up were not consistently documented as part of routine postoperative care during the study period and were therefore not available for analysis. Clinical follow-up focused primarily on symptom assessment and patient-reported outcomes rather than anatomical staging.

Symptom improvement 

Postoperative symptom outcomes were assessed in 86 patients following PFR. A significant improvement in urinary and prolapse symptoms was reported in 72.1% of cases. In contrast, 17.4% of patients experienced persistent symptoms that required ongoing conservative therapy, and 10.5% developed new mild urinary symptoms, such as urgency or frequency. During the available follow-up period (up to 12 months), no patients required reoperation for recurrent pelvic organ prolapse. The difference in symptom distribution was statistically significant (χ²(2, n=86)=58.77, p<0.001). Detailed percentages with 95% CIs are presented in Table 2.

**Table 2:** Postoperative Symptom Outcomes Following Pelvic Floor Reconstruction (n=86) Distribution of postoperative symptom outcomes with 95% confidence intervals and overall p-value from chi-square goodness-of-fit analysis (p<0.001).

Outcome	n (%)	95% CI (lower–upper)	p-Value
Significant improvement	62 (72.1%)	61.8-80.5%	0.0000
Persistent symptoms	15 (17.4%)	10.9-26.8%	
New urinary symptoms	9 (10.5%)	5.6-18.7%	

## Discussion

This analysis supports the safety and effectiveness of PFR among women aged 70 years or older when performed in a structured urogynaecology service. Our absence of intraoperative injuries and low postoperative morbidity aligns with published data, including the series by Tuschy et al. (2012) [8], and is consistent with the BSUG national audit findings [14]. Similar outcomes have been reported in elderly cohorts undergoing vaginal or laparoscopic pelvic floor surgery, where complication rates remain low, and patient satisfaction is high [9-11,16].

Historically, elderly women were often managed conservatively due to perceived surgical risk; however, contemporary data and our results confirm that well-optimised older patients can achieve outcomes comparable to younger women [8]. In a German case-control study comparing women aged ≥80 years with younger controls, the comorbidity burden was higher (CIRS-G 4.1±2.2 vs 2.4±1.7; p < 0.01), yet serious complications (Clavien-Dindo IV/V) were absent [10]. Similarly, a UK laparoscopic series of older patients (mean 82.6 years) reported no perioperative deaths and good anatomical outcomes in 80% of cases [11].

The key determinants of favourable outcomes include rigorous preoperative assessment, optimisation of comorbidities, and multidisciplinary perioperative planning. The high prevalence of obesity and constipation in this cohort reinforces the importance of bowel management and weight optimisation to reduce the recurrence risk [12,14]. Pre-existing hypertension and diabetes did not appear to affect wound healing or recovery, likely due to careful pre-assessment and intraoperative planning, observation consistent with previous literature [17].

The widespread use of vaginal oestrogen, as recommended by NICE [2], may have supported better tissue healing. Although data remain limited, local oestrogen has been associated with improved vaginal tissue quality and potentially enhanced surgical outcomes in postmenopausal women [15]. Anaesthetic technique was individualised (regional or general), enabling early recovery and minimising opioid use, aligning with ERAS protocols in older gynaecology populations [15].

While no reoperations were observed within 12 months, the absence of early reintervention does not exclude later anatomical recurrence, which requires longer-term follow-up and standardised prolapse staging. While early outcomes were excellent, longer-term follow-up should assess recurrence rates beyond 12 months and patient-reported quality-of-life measures. A recent long-term cohort following primary POP repairs demonstrated low reoperation rates over 10 years [12]. Cost-effectiveness analyses may further inform NHS service planning for this expanding population. Nonetheless, our findings reinforce that, when delivered in experienced multidisciplinary settings, surgical intervention in elderly women can be safe, effective, and aligned with modern standards of geriatric surgical care.

A note of caution is warranted for very elderly or frail patients. Some studies indicate increased risk of severe complications among women aged ≥80 years undergoing reconstructive procedures compared with obliterative surgery (OR 2.53; 95% CI 1.01-6.36; p=0.05) [13]. Therefore, patient selection should integrate frailty assessment, geriatric input, and shared decision-making to balance efficacy and safety.

In summary, this study provides context-specific evidence that PFR can be undertaken with low short-term morbidity and meaningful patient-reported functional improvement in carefully selected, well-optimised older women managed within a specialist urogynaecology service. While these findings support the feasibility of offering reconstructive surgery following comprehensive assessment rather than exclusion on the basis of chronological age alone, they should be interpreted within the limits of a single-centre, retrospective design and should not be extrapolated to universal recommendations or non-specialist settings.

Clinical implications

For elderly women with symptomatic POP, age alone should not deter surgical management. A structured preoperative pathway, including frailty assessment, optimisation of cardiovascular and metabolic conditions, preoperative vaginal oestrogen, and early postoperative mobilisation, can minimise morbidity and improve recovery [13,17]. Routine use of validated outcome measures such as the ICIQ ensures standardised evaluation of functional improvement. Our results highlight the importance of keeping access to reconstructive options for well-selected older women, rather than defaulting to conservative management. Over 70% reported improved urinary function and quality of life, consistent with national and international data supporting surgical correction of POP in older women [1,14]. Enhanced mobility, self-esteem, and social participation contribute to improved postoperative well-being [17].

Quality and governance perspective

This project supports adherence to NICE (2021) and BSUG (2022) standards and demonstrates the value of continuous clinical audit in improving patient safety [2,14]. Regular submission to the BSUG national database and use of validated ICIQ questionnaires have strengthened service evaluation and patient feedback mechanisms [14].

Limitations

This study has several important limitations that should be considered when interpreting the findings. As a single-centre retrospective analysis, it is inherently subject to selection and documentation bias, and the results may not be fully generalisable to other institutions with differing patient populations, resources, or surgical practices. Although all procedures were undertaken within a consultant-led specialist urogynaecology service using standardised departmental pathways, unavoidable variation in operative technique and perioperative care between centres may limit direct comparability of outcomes.

While the methodology is described in sufficient detail to support replication of the study design and analytical approach, exact reproducibility across centres is constrained by centre-specific operative protocols and pragmatic outcome definitions typical of quality-improvement work. Formal frailty scoring was not routinely recorded during the study period, limiting the ability to stratify outcomes by physiological reserve or to identify subgroups at differential risk. In addition, no formal adjustment for potential confounding variables was undertaken. Factors such as comorbidity burden, functional status, procedure complexity, and patient preference, rather than random allocation, have influenced outcomes. Given the modest sample size and exploratory audit-based design, multivariable modelling was not feasible, and findings should therefore be interpreted as descriptive rather than causal.

Several outcomes were derived from routine clinical documentation rather than prospectively standardised definitions. Postoperative urinary tract infection was diagnosed based on clinician assessment and/or microbiological confirmation requiring antibiotic treatment; ileus was identified by documented postoperative bowel dysfunction requiring conservative management; and persistent pain reflected ongoing symptoms necessitating follow-up beyond the expected recovery period. While this pragmatic approach reflects real-world clinical practice, it introduces variability in outcome ascertainment and may further limit cross-centre reproducibility.

The sample size was modest, reflecting the relatively low number of elderly women undergoing PFR during the study period. Although complication rates were low and no major intraoperative injuries, returns to theatre, or surgery-related deaths were observed, the absence of rare adverse events in a cohort of this size does not equate to zero risk. Uncommon but clinically significant complications may not be detected without larger multicentre datasets or longer surveillance.

Follow-up was limited to 12 months, precluding assessment of long-term durability and anatomical recurrence. Standardised POP-Q staging was not consistently recorded during routine follow-up, preventing objective anatomical assessment of recurrence. As a result, recurrence outcomes were inferred indirectly through symptom persistence and reoperation rates rather than formal prolapse staging.

Patient-reported outcomes were assessed using available ICIQ data. Although the ICIQ is a validated instrument, incomplete paired score capture limited formal evaluation of numerical score change or MCID. Functional outcome categories were therefore based on patient-reported symptom status rather than predefined PROM thresholds, which may introduce subjectivity but reflect routine clinical practice in this setting. PROM findings should accordingly be interpreted as descriptive rather than inferential.

Future research would benefit from prospective multicentre designs incorporating standardised operational definitions, routine frailty assessment, structured PROM collection with MCID analysis, systematic anatomical follow-up, and longer surveillance for recurrence and reoperation. Such approaches would enhance reproducibility, strengthen causal inference, and allow more precise risk stratification in elderly women undergoing PFR.

## Conclusions

In this single-centre retrospective cohort, PFR in elderly women aged 70 years or older appears to be a safe, effective, and clinically justified intervention and is associated with favourable short-term morbidity and patient-reported functional outcomes in carefully selected, well-optimised patients when delivered within a structured multidisciplinary urogynaecology service. These findings support the feasibility of offering reconstructive surgery to selected older women following comprehensive assessment, rather than excluding patients based on chronological age alone, as optimised women aged ≥70 years can achieve functional outcomes comparable to younger cohorts with low morbidity; rather, outcomes are influenced by comprehensive preoperative assessment, optimisation of comorbidities, and individualised surgical planning.

The study underscores the importance of comprehensive preoperative assessment, optimisation of comorbidities, and adherence to evidence-based national guidelines (such as those endorsed by BSUG and NICE) to ensure favourable results. These findings should be interpreted within the limitations of an exploratory, single-centre cohort. Routine use of multidisciplinary pre-assessment, anaesthetic individualisation, and postoperative rehabilitation further enhances safety and recovery.

From a clinical standpoint, these findings support a paradigm shift away from conservative management by default and reinforce that surgery should be offered based on functional status and patient preference rather than age. As the ageing population expands, establishing dedicated pelvic floor pathways for older women will be vital to maintain quality, equity, and cost-effective care.

However, given the retrospective, single-centre design and limited follow-up, results should not be generalised to all elderly women, nor do they permit causal inference regarding specific perioperative interventions. Future prospective multicentre studies incorporating standardised frailty and functional assessments are required to better define risk stratification, focus on long-term durability, recurrence patterns, and the integration of minimally invasive or robotic-assisted techniques to refine outcomes and quality of life in this growing demographic.
